# A multi-isolate genomic approach identifies diverse Escherichia coli contamination and antimicrobial resistance carriage on retail foods

**DOI:** 10.1099/mgen.0.001549

**Published:** 2025-10-30

**Authors:** Gabriel Astorga, Samuel J. Bloomfield, Raphaëlle Palau, Nicol Janecko, Leonardo de Oliveira Martins, Dipali Singh, Roxana Zamudio, George M. Savva, John Wain, Alison E. Mather

**Affiliations:** 1 Quadram Institute Bioscience, Norwich Research Park, Norwich, NR4 7UQ, UK; 2 Centre for Microbial Interactions, Norwich Research Park, Norwich, NR4 7UG, UK; 3 University of East Anglia, Norwich Research Park, Norwich, NR4 7TJ, UK

**Keywords:** antimicrobial resistance, *Escherichia coli*, food, phylogenetics, population genomics, whole-genome sequencing

## Abstract

**Background.** Bacterial pathogens contaminating retail foods can cause foodborne illness. *Escherichia coli* is commonly enumerated on food to assess unsafe levels of faecal contamination, but *E. coli* as a species is genetically variable and different types of *E. coli* may present more of a health risk than others. This enumeration approach provides limited insight into the types of *E. coli* contaminating food, whereas whole-genome sequencing (WGS) can provide insight into the genetic diversity as well as genes of concern. Current WGS studies have focused on the selection of pathogenic or antimicrobial-resistant *E. coli*, which has provided limited insight into the diversity and potential risk to consumers.

**Methods.** To assess the diversity and potential risk of *E. coli* on food, food samples were collected from retail stores across Norfolk, UK. In this study, 126 chicken, 52 leafy green, 115 pork, 75 prawn and 33 salmon *E. coli*-positive samples were investigated. Up to four *E. coli* were isolated per sample and underwent WGS. *E. coli* genomes underwent *in silico* multi-locus sequence typing, where sequence types (STs) were investigated at the single nucleotide polymorphism (SNP) level. Furthermore, virulence genes and antimicrobial resistance (AMR) determinants were investigated.

**Results.** From the total of 401 food samples, 1,067 *E. coli* genomes were isolated, sequenced and classified into 238 known STs and 54 unknown STs. Of the 145 samples in which four isolates were sequenced, 17 revealed four different STs, suggesting a high within-sample diversity. Within-sample within-ST comparisons revealed up to 845 pairwise non-recombinant SNPs. *E. coli* genomes contained between 0 and 14 AMR determinants. Up to four different AMR determinant combinations within a sample were identified, with 34.7% (*n*=139/401 samples) of all *E. coli*-positive samples containing three or more AMR determinants. In this dataset, 26 putative extraintestinal pathogenic *E. coli* (ExPEC) were identified.

**Discussion.** A multi-isolate WGS approach identified a high diversity of STs, different AMR profiles and putative ExPEC within individual food samples which would have been missed by traditional enumeration approaches. Collecting multiple isolates and WGS is therefore necessary for ensuring thorough microbial hazard characterization for the consumer.

**Impact Statement**

Retail foods can be contaminated with bacterial pathogens that can cause foodborne illness when improperly handled, cooked and consumed. Faecal contamination on food is typically monitored by enumerating coliforms such as *Escherichia coli*. Other traditional *E. coli* monitoring approaches include the identification of specific pathogenic types, or antimicrobial resistant lineages. The general population of *E. coli* contaminating retail foods in the UK remains under-investigated, and traditional approaches do not provide any information on the diversity of *E. coli* to which the consumer is exposed. To address this, we sequenced the whole genomes of 1,067 *E. coli* isolates from a total of 401 food samples which include raw chicken, raw pork, raw and cooked prawns, fresh leafy greens and raw salmon collected at retail stores. We classified them into eight phylogroups and 292 sequence types (STs). When up to four *E. coli* isolates from the same food sample were sequenced, up to four STs with different antimicrobial resistance genotypes were identified. The same STs within the same sample contained up to 845 pairwise non-recombinant single nucleotide polymorphisms (SNPs). These results demonstrate that retail foods are a vector for multiple lineages of *E. coli* that can harbour different antimicrobial resistance determinants. Using a non-selective culturing approach and whole-genome sequencing is useful for the identification of potential retail food microbial threats.

## Data Summary

The sequence data generated in this study are available in the Sequence Read Archive (SRA: PRJNA1177805 and SRA: PRJNA1107692).

## Introduction

Retail foods can be contaminated with a diverse array of micro-organisms [1] which may indicate a lack of hygienic practices in food production, processing and handling [2]. The consumption of pathogens on food can cause gastrointestinal disease [3]. Safe levels of general microbiological contamination on meat and ready-to-eat vegetables have been set in the UK [4], although an estimated 2.4 million cases of foodborne illnesses still occurred in the UK in 2018 [5]. One way pathogens can become present on food is through faecal contamination, which can be monitored by the presence and enumeration of enteric bacteria like *Escherichia coli* [4,6, 7]. Pathogenic *E. coli* lineages, like Shiga-toxin producing *E. coli*, have been a focus of surveillance in the UK [5]. However, most *E. coli* lineages are either commensal organisms or opportunistic pathogens that can cause bloodstream infections [8] and urinary tract infections [9].

Antibiotics can be used to treat bacterial infections, but bacteria like *E. coli* can acquire resistance to these antibiotics, making infections more difficult to treat [10]. *E. coli* is commonly used as a sentinel for antimicrobial resistance (AMR) [11], and AMR was estimated to be involved in 4.95 million deaths globally in 2019, with antimicrobial-resistant *E. coli* being one of the main organisms involved [12]. Although the UK [13,14] and the European Union (EU) [15] regulate and monitor antimicrobial use, there are variable regulations on antimicrobial use in industries of imported food outside these areas [16,17]. The UK relies on global supply chains (EU and non-EU), with 35% of all UK imports (1.2 million tonnes) being food products [18], meaning antimicrobial use in the production of foods consumed in the UK can be variable and may expose the consumer to a range of different levels of food-associated microbial risk [1].

In the event of an outbreak, the UK Health Security Agency (UKHSA) uses biochemical tests to identify *E. coli*, which can be further analysed using whole-genome sequencing (WGS) [2,6]. To identify lineages of concern, *E. coli* can be classified into multilocus sequence types (STs) [19]. WGS is more useful for tracking lineages as it provides higher resolution insight by identifying single nucleotide polymorphisms (SNPs [20]. Short-read WGS has been used previously to track pathogenic lineages, study outbreaks and identify antimicrobial-resistant lineages [19]. However, some studies that use WGS often do not make the number of sequenced * E. coli* per sample clear [21,22].

There is a need to identify potential new risks without being limited to specific lineages. To our knowledge, this is the first study that has taken multiple different samples of raw meats, leafy greens and seafood and used a non-selective, multi-isolate WGS approach for investigating the genomic diversity of *E. coli*. The objectives of this study were to (1) identify the STs and phylogroups that contaminate retail foods, (2) describe the genomic diversity of *E. coli* within different food commodities and within individual samples and (3) estimate the presence of virulence and AMR genes in *E. coli* that may present a health risk to consumers.

## Methods

### Sample collection and culturing

*E. coli*-positive samples were selected from a study described by Janecko *et al*. [1] where a repeated cross-sectional sampling approach was applied in Norfolk, UK, between May 2018 and November 2019 to investigate pathogen contamination on defined food products. In total, 1,369 food products were purchased from 203 chain stores, butcher shops and independent retailers (greengrocers, fishmongers and independent grocers) with the aim of investigating pathogen contamination. The sampling focused on five food commodities that reflected highly consumed food products based on the Family Foods 2015 consumption data [23]. The five food commodities included raw chicken, raw pork, raw/cooked prawns, raw salmon and pre-packaged leafy greens [1].

Sample processing, preparation, *E. coli* microbiological detection and isolation were previously described in Janecko *et al*. [1]. In summary, food samples were processed within 24 h of purchase and 100 g of the sample was transferred into stomacher bags, whilst maintaining a sterile chain. All samples were homogenized in 225 ml of buffered peptone water (BPW) [Southern Group Laboratory (SGL), Corby, UK] using a Stomacher® bag at 100 r.p.m. for 30 s (Seward stomacher 400C laboratory blender, Worthing, UK) and then incubated at 37 °C for 24±3h.

Once incubated, 50 ml of the BPW was added to 50 ml of *E. coli* enrichment broth double concentration (EC 2×) (ThermoFisher Diagnostics, Rochford, UK) and incubated at 42 °C for 24±3h. Eosin methylene blue agar (Sigma-Aldrich, Haverhill, UK) was inoculated with 10 µl loops of the incubated broth and incubated at 37 °C for 24±3h; all subsequent agar plates were incubated for the same time and temperature. Up to four colonies that exhibited typical *E. coli* morphology (dark maroon colony with a green sheen) were selected for further subculturing. These colonies are hereafter termed isolates. Each isolate was subcultured on MacConkey agar (ThermoFisher Diagnostics) and finally subcultured onto tryptic soy agar (Trafalgar Scientific Ltd., Leicester, UK). Isolates were biochemically confirmed to be *E. coli* by a negative result using Simmon’s citrate agar (Sigma-Aldrich) and a positive result using Remel indole spot reagent test (Fisher Scientific, Loughborough, UK).

In the original cross-sectional study [1], there were 859 *E. coli*-positive samples, each with up to four *E. coli* colonies stored at −70 °C in 1 ml of Brucella broth +17.5% glycerol (ThermoFisher Diagnostics) [1]. From these, 401 *E. coli*-positive samples with up to four *E. coli* isolates per sample were used in this study, with a preference for those samples that yielded four isolates. Out of these 401 samples, a batch of 109 was previously used to assess host DNA depletion methods for food metagenomes [24] and deposited in the Sequence Read Archive under BioProject PRJNA1107692 [25]. The approach for sample selection for sequencing is further described in Method S2 (available in the online Supplementary Material).

### Whole-genome sequencing

Genomic DNA was extracted from *E. coli* isolates using Promega Maxwell RSC Cultured Cell DNA kits (Promega, Southampton, UK) according to the manufacturer’s instructions. Libraries were formed using Nextera XT library preparation kits (Illumina, San Diego, CA, USA) and sequenced on an Illumina NextSeq system to create 150 bp Illumina paired-end reads. Whole-genome sequenced isolates are hereafter referred to as genomes. Genomes were deposited in the Sequence Read Archive under BioProject PRJNA1177805 (Table S1).

### Quality control and assembly

Genomic analyses were performed on the high-performance computing cluster at the Norwich Bioscience Institutes, Galaxy [26] and a Quadram Institute Bioscience QIB-CLOUD Virtual Machine (adapted from the Cloud Infrastructure for Microbial Bioinformatics) [27]. Raw read adapter sequences were trimmed using Trimmomatic v.0.33 [28], assembled using SPAdes v.3.1.1 [29] using k-mer lengths 21, 33, 55 and 77 and the ‘careful’ parameter, and assembly quality was analysed using QUAST v.4.6.3 [30] and by aligning the trimmed reads to the genome assemblies (details of tools used to calculate mean read depth are available in Method S1). To check for contamination, CheckM v.1.0.11 [31] was used to identify duplicate genes, and genomes with >50 were excluded. The average nt identities of the genome assemblies were compared to *E. coli* K-12 substr. MG1655 [32] (GenBank accession assembly ID: GCA_000005845.2) using FastANI v.1.32 [33]. *E. coli* genomes passed quality control if they were between 4.4 and 5.7. Mbp in length, had an average GC content between 42.6–54.6 mol% [34] and had a mean read depth of their four largest contigs above 28.

### *E. coli* phylogroup, sequence typing and phylogenetic analysis

*E. coli* genome assemblies were classified into phylogroups using Clermontyping v.20.03 [35] and into STs using MLST v.2.19.0 [36] with the *E. coli* PubMLST seven-gene scheme (https://github.com/tseemann/mlst). Unclassified STs were further characterised into matching unknown STs by identifying unique combinations of *adk*, *fumC*, *gyrB*, *icd*, *mdh*, *purA* and *recA* profiles provided by the MLST output. Phylonium v.1.6 [37] with default settings was used to estimate the phylogenetic distances between the * E. coli* assemblies. A dendrogram was estimated using rapidNJ v.2.3.2 [38] and was visualised using R v.4.1.3 [39]; further phylogeny parameters and R packages are detailed in Method S3.

### Pairwise SNP analysis

The reads from the three most abundant STs in this collection were selected to assess within-sample within-ST diversity via pairwise SNP differences. Reference genomes for these three STs were chosen using Referenceseeker v.1.8.0 [40] and the bacteria RefSeq database (downloaded January 2024) [41]. Genomes were grouped into their STs. References with the highest average nt identity and highest conserved DNA, as determined by Referenceseeker v.1.8.0, that were representative of the majority of the genomes (>50% of genomes in the dataset) were chosen as the reference for that ST.

Reads from each ST of interest were aligned to their respective reference whole-genome assembly [ST10 reference (GenBank accession assembly ID: GCA_900636145.1), ST101 reference (GenBank accession assembly ID: GCA_029318875.1) and ST117 reference (GenBank accession assembly ID: GCA_025369795.1)] using Snippy v.4.6.0 (https://github.com/tseemann/snippy). Gubbins v.3.2.0 [42] was used to remove SNPs attributed to putative recombination from the alignments. The pairwise SNP differences were calculated using SNPdist v.0.7 (https://github.com/tseemann/snp-dists). Genetically similar STs within the same sample were defined as genomes that differed by five or fewer SNPs from the reference, as this was the UKHSA threshold used for an outbreak of Shiga toxin-producing *E. coli* O157 [20].

### Diversity between food commodities

The *E. coli* ST diversity (richness and inverse Simpson index) was estimated for each food commodity. The R package iNEXT v.3.0.1 [43] rarefied and extrapolated the data to predict and compare diversity between food commodities. Richness (*q*=0) and inverse Simpson index (*q*=2) were calculated using the ‘incidence_freq’ datatype, with confidence intervals set to 95% and the number of bootstraps set to 500.

### AMR genes, plasmid replicons and virulence genes

ARIBA v.2.14.6 [44] with default settings on the trimmed reads and the ResFinder [45] (downloaded in May 2023) was used to identify acquired AMR genes. Known point mutations associated with AMR were identified using PointFinder (python script downloaded in October 2024) (https://github.com/guthrielab/pointfinder) [46] and the PointFinder database (downloaded in October 2024) [46]; for this analysis, we focused on acquired genes or point mutations with strong evidence of phenotypic resistance. Identified acquired AMR genes and point mutations were collectively called AMR determinants. AMR determinants were categorised into their antimicrobial classes (Method S4, Tables S2 and S3) and were prioritised according to the World Health Organization prioritisation scheme [47]. Potential multidrug resistance (MDR) in this study was classified as the presence of AMR determinants conferring resistance to three or more different antimicrobial classes according to the WHO antimicrobial class list [47,48].

Plasmid replicons and virulence genes were determined using ARIBA [44] with default settings on the trimmed reads, with the PlasmidFinder [49], and VFDB (virulence factor database) core [50] databases. Typical genetic markers for diarrhoeagenic * E. coli* were investigated: enteropathogenic *E. coli – eae *and *bfp*; Shiga-toxin producing *E. coli*/verocytotoxigenic *E. coli – stx1* and *stx2*; enterotoxigenic – *elt* and *estA/estB*; enteroinvasive *E. coli* – pINV; enteroaggregative – pAA; diffuse adhering *E. coli* – Afa/Dr family [51]. *E. coli* was also characterised as extraintestinal pathogenic (ExPEC) if two or more of the following virulence genes were present in the genome: *papAH*, *papC*, *sfa/focDE*, *afa/draBC*, *iutA* and *kpsMII* [52]. AMR genotypic profiles were defined as the unique combination of AMR genes and point mutations for each genome.

## Results

### *E. coli* contamination on food

A total of 1,067 *E. coli* isolates recovered from chicken (*n*=310 isolates), leafy greens (*n*=162 isolates), pork (*n*=279 isolates), prawns (*n*=206 isolates) and salmon (*n*=110 isolates) underwent WGS and passed quality control (Table 1).

**Table 1.** Percentage of samples and total number of isolates (*n*) across each food commodity purchased in Norfolk, UK, between May 2018 and November 2019

Commodity	Percentage of samples with 1 isolate	Percentage of samples with 2 isolates	Percentage of samples with 3 isolates	Percentage of samples with 4 isolates	Total no. of samples	Total no. of isolates
Chicken	17.5 (*n*=22)	46.8 (*n*=118)	7.94 (*n*=30)	27.8 (*n*=140)	126	310
Leafy greens	13.5 (*n*=7)	17.3 (*n*=18)	13.5 (*n*=21)	55.8 (*n*=116)	52	162
Pork	13.9 (*n*=16)	54.8 (*n*=126)	6.09 (*n*=21)	25.2 (*n*=116)	115	279
Prawn	6.67 (*n*=5)	52.0 (*n*=78)	1.33 (*n*=3)	40 (*n*=120)	75	206
Salmon	6.06 (*n*=2)	21.2 (*n*=14)	6.06 (*n*=6)	66.7 (*n*=88)	33	110

The 1,067 *E. coli* genomes were classified into 238 known STs and 54 unknown STs (Table S4). The five most abundant STs combined accounted for 19.5% of the total dataset (Table S5, Fig. S1). Chicken (*n*=118 STs) and pork (*n*=103 STs) samples had the highest number of unique STs, followed by prawns (*n*=75 STs) and then leafy greens (*n*=68 STs), and salmon samples had the lowest number of unique STs (*n*=22 STs) (Table S6).

### Diversity estimates across commodities

**Fig. 1.**
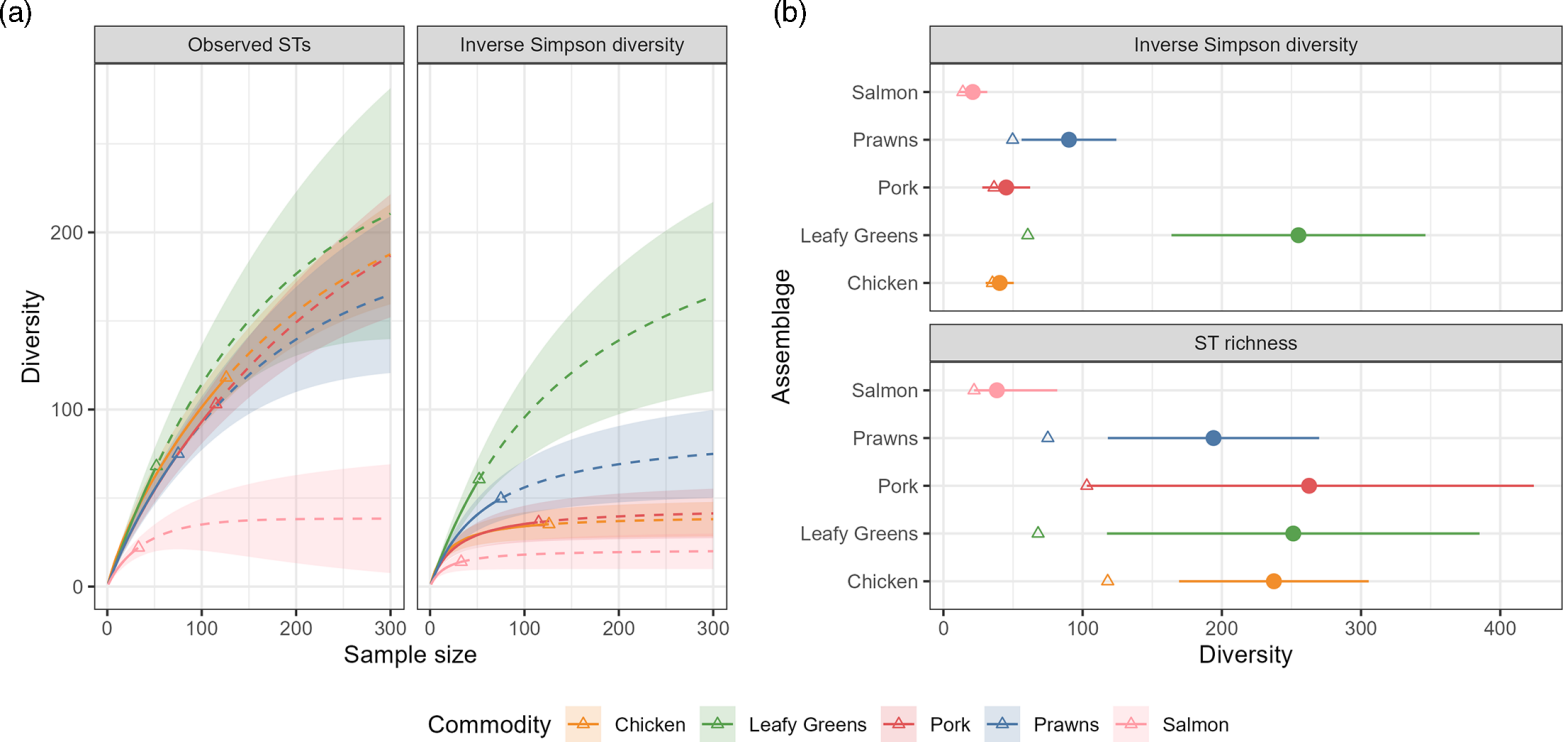
**(a**) The rarefaction curves and the extrapolated diversity indices for the STs on each commodity with bootstrapped 95% confidence intervals. Points represent the observed number of distinct STs and the number of samples collected. Solid lines are rarefaction curves, and dotted lines represent extrapolations. Shaded areas are 95% confidence intervals based on bootstrapped standard errors. (**b**) The asymptotic diversity indices (dots with 95% confidence intervals, along with the observed diversity [triangles] for each commodity) coloured by food commodity.

The estimated richness appeared similar across all commodities except salmon, which had a smaller number of observed and estimated STs compared with the other commodities. The Simpson’s diversity of leafy greens was higher than the other commodities, reflecting a more even ST distribution when compared to the other food commodities (Fig. 1a, b). Of the 68 distinct STs observed in 52 leafy green samples, only nine STs were observed in two different samples and only one ST in three different samples. However, in both pork and chicken, there were STs that were common to many more samples, for example, ST10 (*n*=20) and ST101 (*n*=14) being found in many of the 115 pork samples and ST117 (*n*=29) and ST10 (*n*=18) seen in many of the 126 chicken samples.

### *E. coli* ST diversity within samples

A total of 48.1% of samples (*n*=193/401) contained two or more *E. coli* STs, and 14.0% (*n*=56/401) of samples were contaminated with three or more STs. Salmon samples were the only food commodity observed to be contaminated with a maximum of two *E. coli* STs within one sample (Fig. S2).

**Fig. 2.**
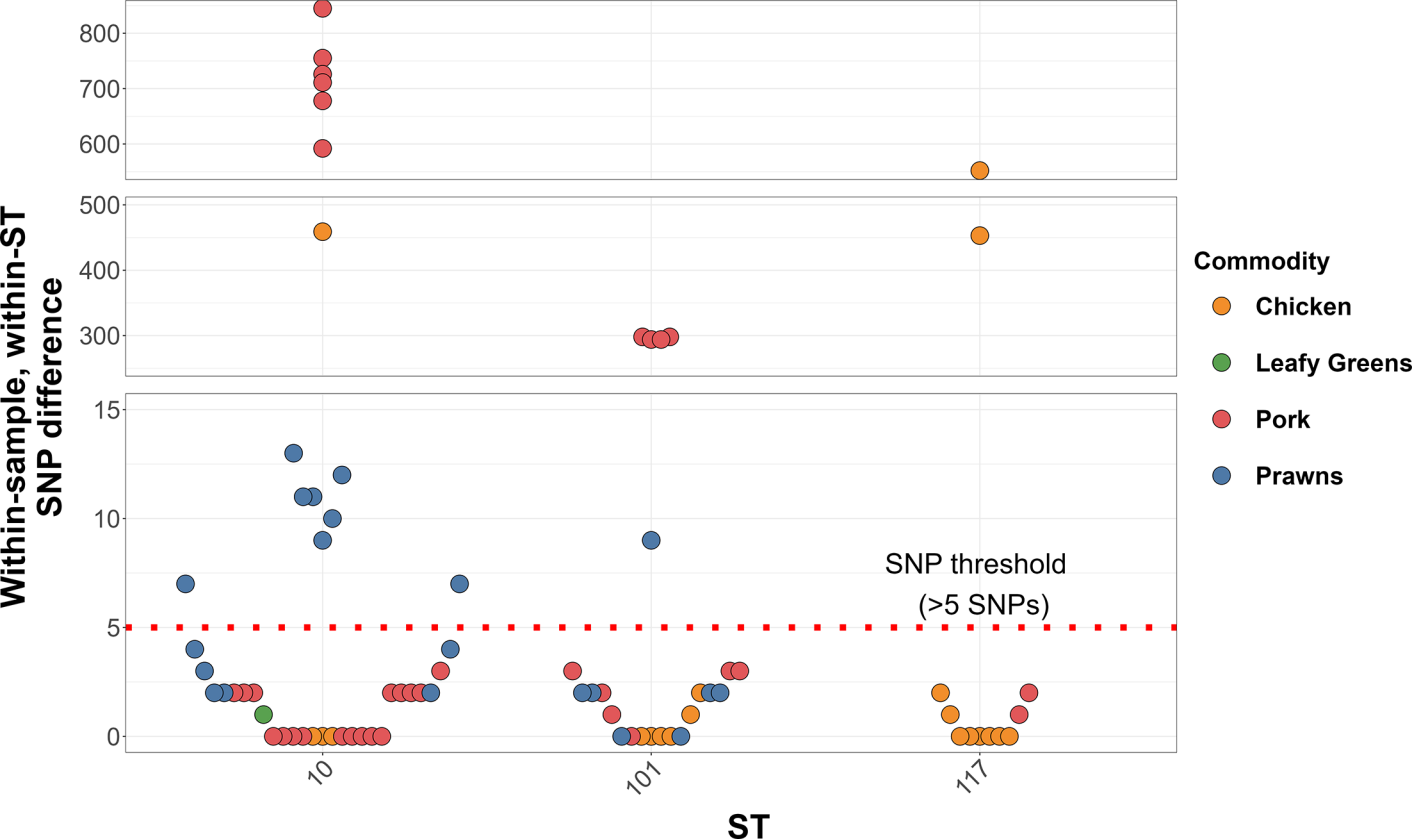
Pairwise comparisons of the within-sample within-ST SNP differences across ST10, ST101 and ST117 within the collection of 1,067 *E. coli* genomes isolated from retail chicken, pork, prawns, salmon and leafy greens. Within-sample within-ST comparisons are coloured by the food commodity from which they were isolated. The five SNP thresholds for epidemiologically linked *E. coli*, as used by the UKHSA in investigation of a Shiga toxin-producing *E. coli* O157 outbreak [20], are indicated by the red dotted line.

The three most abundant STs were ST10 (*n*=73 genomes), ST117 (*n*=41 genomes) and ST101 (*n*=38 genomes). There were 34 samples which were contaminated with at least two ST10s (*n*=18 samples), ST117s (*n*=8 samples) or ST101s (*n*=8 samples). There were 77 pairwise ST comparisons within the same sample from these three STs. In 22 of these comparisons, the SNP distances between genomes within the same sample and ST exceeded the five SNP threshold, reaching a maximum of 845 pairwise SNPs (Fig. 2). Salmon was the only food commodity from which multiple contaminating ST10s, ST101s or ST117s were not recovered from the same sample.

### *E. coli* plasmid replicons, virulence genes and AMR genes

In this dataset, 63 unique plasmid replicon types were identified, with the three most frequent belonging to the IncF incompatibility type (Fig. S3). The median number of different plasmid replicons in each genome was 2 (range=0–9). No typical intestinal pathogen (InPEC) genetic markers *stx1*, *stx2*, *eae*, *elt*, *estA/estB*, *bfp* or Afa/Dr family were found in this dataset. The extraintestinal pathogenic *E. coli* (ExPEC) virulence factor scheme identified 26 putative ExPEC across 17 samples from chicken (*n* samples=10, genomes=13) and pork (*n* samples=7, genomes=13). Putative ExPEC belonged to eight known and two unknown STs; ST117 had the highest number of genomes classified as putative ExPEC (*n* samples=7, genomes=10).

In this dataset, 58.3% genomes (*n* genomes=622, *n* samples=261) had no AMR determinants detected. There were 176 samples where all *E. coli* genomes had no AMR determinants identified. At least one AMR determinant was found in 66.1% of *E. coli* genomes from chicken, 47.0% of genomes from pork, 31.6% genomes on prawns, 18.2% genomes on salmon and 14.8% of genomes on leafy greens. The AMR determinants were classified into 13 different antimicrobial drug classes, which included eight critically important antimicrobial drug classes and five highly important drug classes (Table 2).

**Table 2.** The total sample counts and percentages of the collection of 401 food samples consisting of 1,067 *E. coli* genomes isolated from retail chicken, pork, prawns, salmon and leafy greens carrying AMR determinants. The AMR determinants are characterised into antimicrobial classes. Clinical importance was determined using the World Health Organization prioritisation categorization

AMR class	No. of samples with AMR determinants (%)	World Health Organization prioritization categorization of clinically important antimicrobials
Beta-lactam	145 (36.2)	Critically important
Aminoglycoside	141 (35.2)	
Quinolone	83 (20.7)	
Phosphonic acid derivatives (fosfomycin)	11 (2.74)	
Macrolide	9 (2.24)	
Oxazolidinone	5 (1.25)	
Polymyxins (colistin)	2 (0.499)	
Anamycins (rifamycin)	1 (0.249)	
Tetracycline	146 (36.4)	Highly important
Sulphonamide	133 (33.2)	
Trimethoprim	120 (29.9)	
Chloramphenicol	46 (11.5)	
Lincosamide	18 (4.49)	

In this study, 158 *E. coli*-positive samples (39.4%) were contaminated with potential MDR *E. coli*. Samples containing at least one MDR genome were found across all food commodities and were observed in 60.3% of chicken samples, 35.7% of pork samples, 22.7% of prawn samples, 6.06% of salmon samples and 5.77% of leafy green samples. The proportion of samples containing MDR isolates was significantly different between food commodities ($X^{2}$ = 72.5, d.f. = 4, *P* = 6.66×10^−15^).

**Fig. 3.**
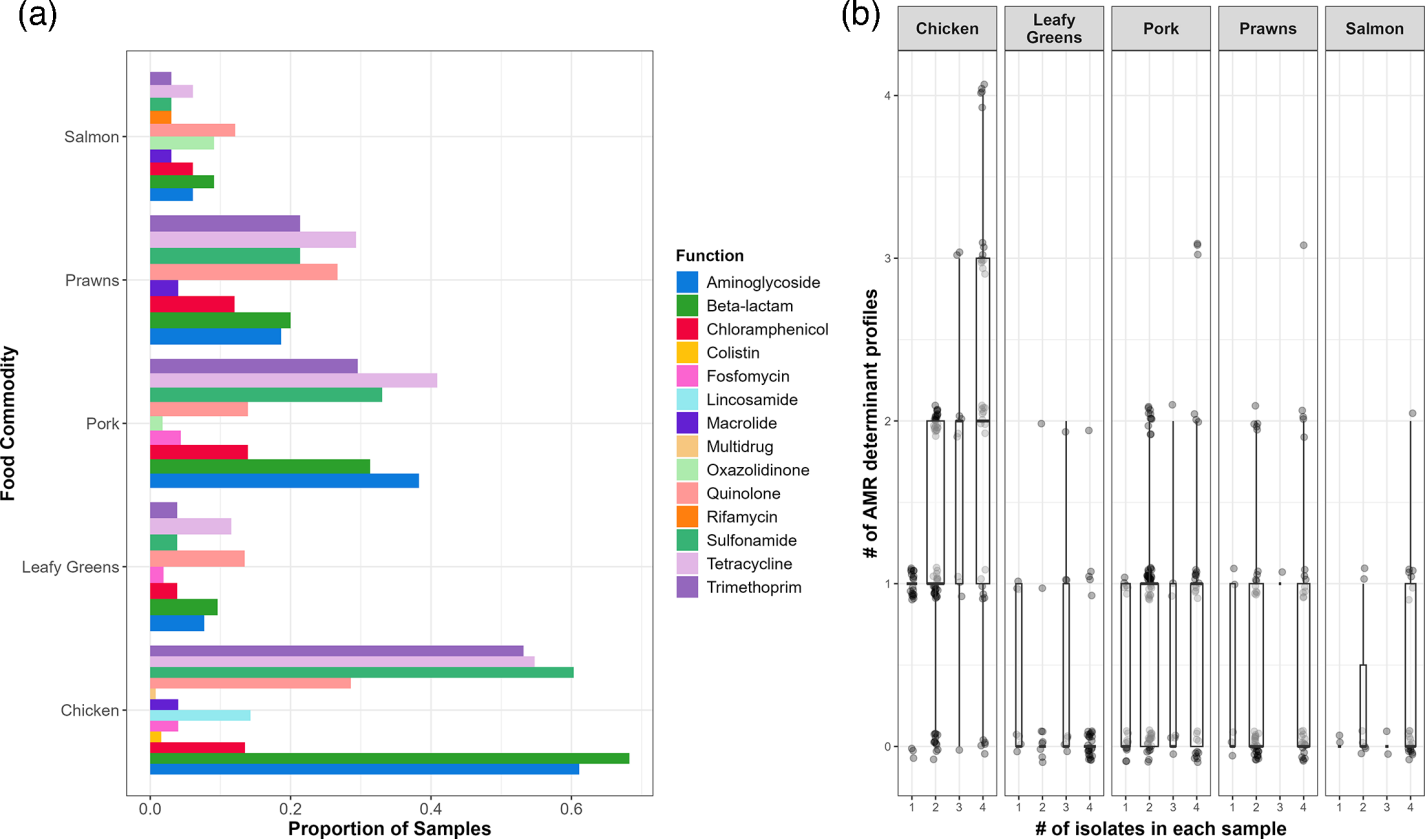
The AMR determinants found within the collection of 1,067 *E. coli* genomes isolated from a total of 401 retail chicken, pork, prawns, salmon and leafy green samples. (**a**) The proportion of samples for each food commodity containing at least one AMR determinant within each food commodity. AMR determinants classed into AMR drug class. (**b**) Boxplots of the 1,067 *E. coli*, showing the number of unique combinations of AMR determinants (termed AMR genotypic profiles) recovered in all 401 food samples when up to four *E. coli* genomes were sequenced, separated by food commodity. Each dot is one sample.

*E. coli* from chicken meat in this study contained AMR determinants which potentially confer resistance to 12 drug classes and one gene [*erm*(42)] that may confer resistance to multiple classes. *E. coli* isolated from salmon samples contained AMR determinants to ten drug classes; pork samples had AMR determinants to nine drug classes; leafy greens and prawns had AMR determinants to eight drug classes (Fig. 3a). Additionally, when up to four isolates per sample were sequenced, up to four different AMR genotypic combinations were often found (Fig. 3b).

### Phylogeny of *E. coli* contaminating retail foods

**Fig. 4.**
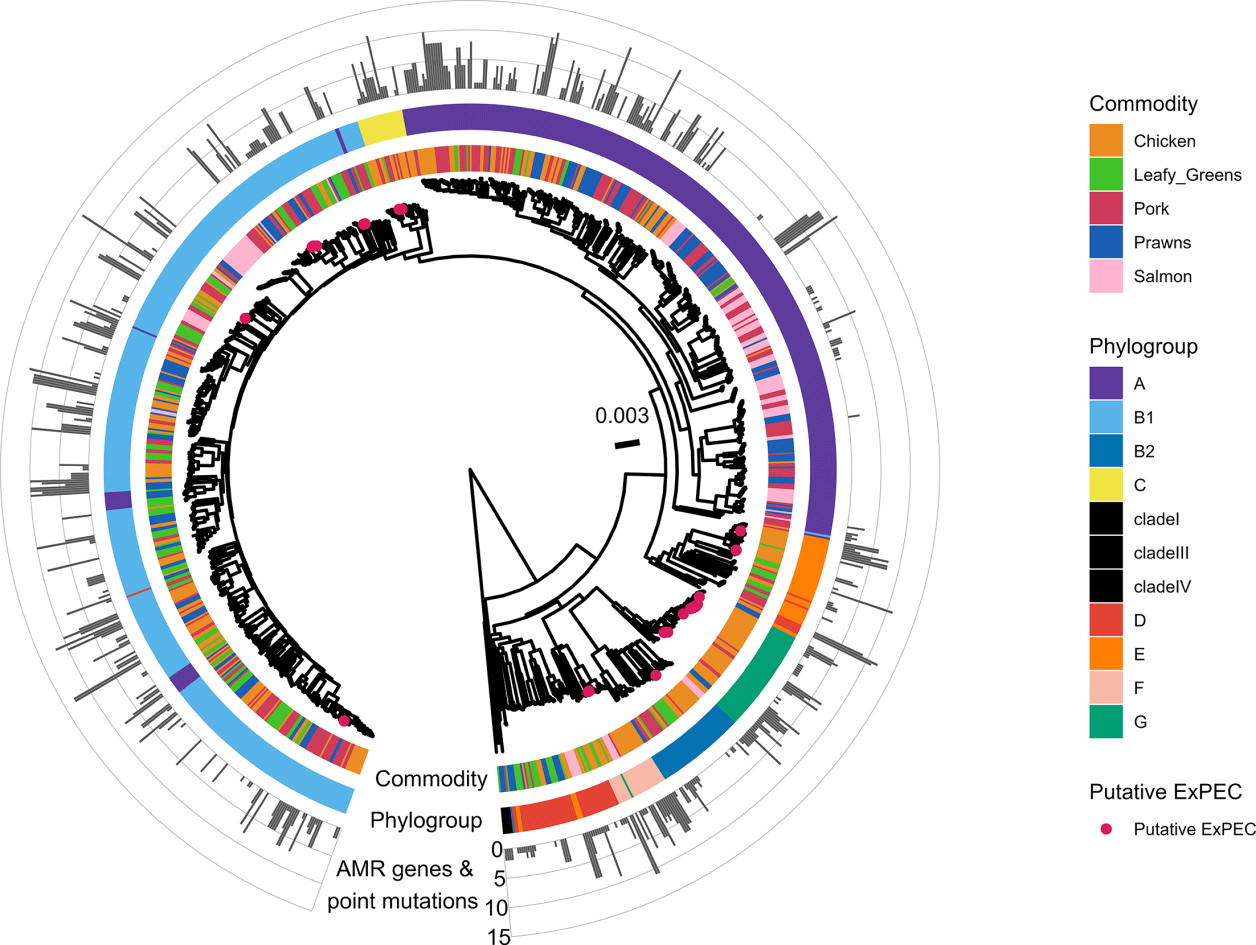
Phylonium midpoint rooted phylogenetic tree of the collection of 1,067 *E. coli* genomes isolated from retail chicken, pork, prawns, salmon and leafy greens with a tree scale estimating 0.003 nt substitutions per site. Tree tips are labelled red if they are putative ExPEC. The commodity (inner ring), phylogroup (middle ring) and the total number of AMR determinants are mapped onto the tree (outer ring).

The *E. coli* phylogenetic tree was divided into phylogroups A, B1, B2, C, D, E, F and G. *E. coli* genomes classified into phylogroups B1 (40.9%) and A (34.0%) comprised the majority of the collection (Table S7). Phylogroups A, B1, B2 and D contained *E. coli* from all food commodities (Figs S4–S6). The 26 putative ExPEC were identified across phylogroups G (*n*=11), B1 (*n*=8), E (*n*=2), F (*n*=2), C (*n*=2) and B1 (*n*=1), and between 0 and 14 unique AMR determinants were identified within individual genomes across the whole dataset (Fig. 4).

There were two clades in particular which were predominantly associated with single food commodities. Phylogroup G was identified to have 53 genomes from 39 samples, which were classified into 10 unique STs. The majority of phylogroup G was comprised of *E. coli* isolated from chicken (*n* genomes from chicken=44/53, *n* chicken samples=34/39), and the majority of genomes were classified as ST117 (*n* genomes classified as ST117=41/53). The number of AMR determinants in each genome from phylogroup G ranged from 0 to 8, and 28 samples had *E. coli* with at least one AMR determinant (Fig. S7). A subclade of phylogroup B1 was comprised entirely of *E. coli* isolated from salmon (*n* genomes=19; *n* samples=7) and was classified as ST5474. These genomes had no AMR determinants detected (Fig. S8).

## Discussion

In this study, using WGS on up to four *E. coli* isolates per sample revealed a diverse population of *E. coli* contaminating retail foods with different combinations of AMR determinants. Relying solely on enumeration is insufficient for risk assessment as the severity of infection and the infectious dose depend on the type of *E. coli* present [53]. Therefore, the use of a WGS approach provides genomic information and insight into virulence genes or AMR determinants, which can add more information for public health risk assessment.

This study classified 1,067 genomes from 401 samples into 238 known and 54 unknown STs across five retail food commodities. Retail chicken (*n* samples=126, *n* STs=118) and pork (*n* samples=115, *n* STs=103) in this study were contaminated with higher numbers of different STs compared to previous work in China, which examined more samples, but sequenced only one *E. coli* isolate per sample from chicken (*n* samples=142, *n* STs=84) and pork (*n* samples=188, *n* STs=81) [54]. This highlights the necessity for typing multiple isolates, as one isolate is not representative of the contaminating STs of *E. coli* on these meats. Multiple STs per sample were also observed on leafy greens (*n* samples=52, *n* STs=68 STs) but are not necessarily comparable to previous studies that selected for tetracycline-resistant *E. coli* on leafy produce [55]. There is currently a lack of WGS studies investigating *E. coli* contamination on seafood, but one study selecting for tigecycline-resistant *E. coli* on fish (*n* samples=7), shrimp and clam (*n* sample=1) classified nine *E. coli* into eight different STs [56]. The lack of *E. coli* surveillance on leafy greens and seafood highlights this current research as important for future risk assessment and surveillance programmes across these food commodities.

The presence of four unique STs when up to four *E. coli* were taken per sample suggests that the *E. coli* contaminating individual samples exist in a heterogeneous population. This has been previously observed using a lower resolution Enterobacterial Repetitive Intergenic Consensus Polymerase Chain Reaction approach, which identified up to five distinct genotypes contaminating individual chicken and pork meat samples [57]. Furthermore, whilst there were a limited number of clades specific to a single food commodity, overall food commodities were spread across the phylogenetic tree. The diverse *E. coli* population surviving and potentially thriving on foods at the retail level indicates successful contamination events, possibly from a combination of faecal contamination by the hosts [58,59], the workers handling the food and/or the processing environment [60]. Leafy greens differ as the contamination events may not necessarily come directly from an animal host, but rather from contaminated irrigation water, or manure used as fertilizer [61]. This as well as the variety of different leafy greens in this study may be a reason for the higher inverse Simpson’s diversity for this commodity compared to the others. The diversity results from salmon, on the other hand, indicate that salmon in this study were less diverse than the other food commodities and we likely captured the full ST diversity present.

A number of clades were less diverse, including phylogroup G, which was comprised predominantly of genomes from chicken meat. The majority of these were classified as ST117, which is a globally distributed potential ExPEC lineage associated with poultry and often found on chicken meats [62,64]. The variability of AMR determinants within these ST117 genomes in this study and their potential as putative ExPEC highlights these *E. coli* as clones of concern for human health. A salmon-specific clade in phylogroup B1 was also identified but did not contain any AMR determinants. The presence of *E. coli* on farmed fish is thought to be influenced by environmental factors like water temperature and pH [65], and for wild-caught fish, the fishing vessel can be another potential point of contamination introducing different lineages of *E. coli* [66].

These contamination sources and events may introduce multiple *E. coli* lineages, which can be further distinguished at the SNP level. Using the three most common STs in this dataset as exemplars to investigate SNP diversity within the same ST on the sample, there were 22 cases where the same STs contaminating a sample had greater than five pairwise nonrecombinant SNP differences, which exceeds a previously used threshold for clonal *E. coli* O157 outbreaks [20]. Previous work on *E. coli* ST131 estimated 2.7 SNPs accumulating per year per genome [67]. Food processing can take days or weeks; therefore, it is unlikely that the same ST within the same sample accumulated these SNPs through persistence in the food processing chain. To note, the stringent reference-based approach used here to investigate intra-ST/intra-sample SNPs will likely underestimate the number of SNPs between genomes and introduce some bias due to the genetic variability within STs [68]. There are also multiple methods available to construct phylogenetic trees, including reference-based approaches and the pairwise distance-informed phylogeny used to assess genetic relatedness across the entire collection of 1,067 genomes. Whilst different methods may result in slightly different tree topologies and each has its advantages and disadvantages, all enable an understanding of the diversity of *E. coli* within this dataset.

The *E. coli* strains we sequenced likely pose a low direct intestinal pathogenic risk to consumers as no *stx1*, *stx2*, *eae*, *bfp*, *let*, *estA/estB* or the Afa/Dr family virulence genes were detected. However, defining intestinal pathogenic *E. coli* based on virulence genes alone is challenging. *E. coli* pathotype hybrids blur the definition of existing distinct *E. coli* pathotypes [69], and *E. coli* with pathogenic-associated virulence genes may not display symptoms in a host [70]. A virulence gene scheme by Johnson *et al*. has been defined for ExPEC [52], which, when applied to this dataset, classified 26 genomes from chicken and pork as putative ExPEC. Contaminated retail foods have been suggested to be a vehicle for ExPEC [57], and there is a risk to consumers as they are constantly exposed to foods. The absence of detectable putative ExPEC on leafy greens, salmon and prawns does not exclude them as potential vehicles for pathogenic lineages; leafy greens can be a vehicle for pathogenic *E. coli* [20], and may present a potentially greater risk than the other food commodities as leafy greens are typically eaten raw and may not be washed before consumption.

We found AMR determinants in 448 genomes (42.0%) across 225 *E. coli*-positive samples. Whilst we focused on acquired AMR genes and point mutations known to confer phenotypic resistance, there can be other AMR determinants, such as point mutations leading to overexpression of efflux pumps [71], which would not be captured by our approach. Different bioinformatic pipelines can also influence the number and type of AMR determinants identified, highlighting the importance of curated databases and harmonisation of typing methods [72,73]. Using our approach, the most prevalent WHO critically important resistance class to which determinants were found in this dataset was beta-lactams. In this study, *bla*_CMY-2_, *bla*_CTX-M-27_ and *bla*_CTX-M-55_ associated with extended-spectrum cephalosporin resistance [74] were identified, which aligns with previous work suggesting retail meats as reservoirs for extended-spectrum beta-lactamase-producing bacteria dissemination [19,21]. This risk is also elevated by potential MDR *E. coli*, which were identified in 33.4% of samples within this dataset. AMR genes can be mobile and have been shown to spread between different *E. coli* phylogroups [75] and across species to bacteria such as *Salmonella enterica* Typhimurium [75] and *Klebsiella pneumoniae* [76]. Previous work investigating microbial communities estimated that retail foods can be contaminated with varying concentrations of AMR genes [24]. This is important as *in vitro* gut model studies have identified that consumption of high bacterial loads with AMR genes can survive the digestive process and spread AMR determinants [77,78]. Whilst many of the foods examined here would be cooked prior to consumption which would lower the AMR gene burden, some foods, such as leafy greens, are usually consumed raw and thus pose a different health risk.

This study shows current approaches for assessing consumer risk for *E. coli* contamination can benefit from the additional genomic information provided from this multi-isolate WGS approach. WGS is still not utilised as a first-choice comprehensive screening system in many countries and is used as a response for outbreaks or for identifying sentinel organisms. The use of WGS within this work shows potential for future use in conjunction with current food safety policies [79]. Further studies can find the optimal number of isolates, which can then be applied to future screening approaches. Additionally, due to the complex nature of the food chain, the contamination observed will be difficult to be attributed to one source without thorough examination of the original host or environmental source, processing and distribution facilities and retail environments.

## Conclusion

Consumers are potentially exposed to a multitude of *E. coli* lineages with different food commodities and individual samples presenting with different levels of AMR risk. Whilst putative MDR *E. coli* was identified in this study, the majority of genomes did not harbour AMR determinants. As this work indicates, taking one isolate as a representative for a sample or selecting for specific AMR may underestimate the diversity of *E. coli* contamination on food. Further work is required to assess *E. coli* contamination at the retail level at an international scale as food importation and globalisation of the food chain increases. A One Health approach, considering host animals, food processing environments and humans, is essential for understanding the dynamics of *E. coli* and foodborne pathogen contamination. To develop further current safety policies, a WGS multi-isolate approach for surveillance is needed in addition to current approaches to monitor public health risks.

## Supplementary material

10.1099/mgen.0.001549 Uncited Table S1.

10.1099/mgen.0.001549 Uncited Supplementary Material 1.

## Abbreviations

AMR: antimicrobial resistance
BPW: buffered peptone water
EU: European Union
ExPEC: extraintestinal pathogenic *Escherichia coli*
MDR: multidrug resistance
ST: sequence type
UKHSA: UK Health Security Agency
WGS: whole-genome sequencing
