# Supplementary material for: A multi-isolate genomic approach identifies diverse Escherichia coli contamination and antimicrobial resistance carriage on retail foods
Source: Microb Genom. 2025 Oct 30;11(10):001549. doi: 10.1099/mgen.0.001549 (PMC12574842; doi:10.1099/mgen.0.001549)
Supplement: Uncited Supplementary Material 1. [file mgen-11-01549-s002.pdf]

Supplementary Materials:

Supplementary Method S1. Quality Control

The trimmed reads were aligned to the genome assemblies using the Burrows-Wheeler aligner v.0.7.17 [1], and analysing the read depth and coverage using SAMtools v1.9 [2] and bcftools v.18 by SAMtools [2] using default parameters.

Supplementary Method S2. Sequencing approach for sample batches

Prior to the start of this project, 516 *Escherichia coli* isolates from 197 samples were selected for WGS. An initial collection of 516 *E. coli* isolates from 197 *E. coli*-positive samples were sequenced before the start of this project. The samples were selected for four reasons.

1) Chicken (n = 15/15 *E. coli*-positive samples), leafy greens (n = 7/15 *E. coli*-positive samples), pork (n = 10/15 *E. coli*-positive samples), prawns (n = 6/9 *E. coli*-positive samples) and salmon (n = 2/9 *E. coli*-positive samples) samples were selected to confirm that the culture method was accurate for detecting *E. coli*. Up to a maximum of four isolates with different morphologies were taken from each *E. coli* positive sample.

2) Chicken (n = 28/28 *E. coli*-positive samples), leafy greens (n = 18/24 *E. coli*-positive samples), pork (n = 14/15 *E. coli*-positive samples), prawns (n = 3/18 *E. coli*-positive samples), and salmon (n = 5/24 *E. coli*-positive samples) samples were previously used to assess host depletion methods for food metagenomes [3] and deposited in the Sequence Read Archive under project PRJNA1107692. Up to a maximum of four isolates with different morphologies were taken from each *E. coli* positive sample.

3) Prawn samples (n = 57/175 *E. coli*-positive samples) were chosen to investigate *E. coli* contamination on prawns, where up to a maximum of two isolates were chosen for each *E. coli* positive sample.

4) Chicken (n = 10 *E. coli*-positive samples), leafy greens (n = 8 *E. coli*-positive samples), pork (n = 4 *E. coli*-positive samples) and salmon (n = 10 *E. coli*-positive samples) samples, where up to a maximum of two isolates were randomly chosen for each *E. coli* positive sample.

To explore further the diversity of *E. coli* contaminating retail foods, samples with four isolates were prioritised for further sequencing. In total, 401 *E. coli* positive samples with up to four *E. coli* isolates per sample were used in this study. The 401 samples included 126 raw

chicken samples, 115 raw pork samples, 75 raw and cooked prawns, 52 raw leafy green samples and 33 raw salmon samples.

Supplementary Method S3. Phylogenetic analysis

The output from Phylonium v.1.6 was input into rapidNJ v.2.3.2 [4] with the -i pd -o t -x options and the phylogenetic tree was visualised with R v.4.1.3 [5] in RStudio v. 2021.09.1 [6] using the ggtree v.3.2.1 [7], ape v.5.5 [8], phytools v.1.0-1[9], and ggnewscale v.0.4.5 [10] packages. Plots were visualised using ggplot2 v. 3.3.5 [11].

Supplementary Method S4. Antimicrobial classification

The output of ARIBA v.2.14.6 [12] with the Resfinder database [13] (downloaded May 2023) provided a gene name and an NCBI accession code, which allowed tracking of the associated sequence in the repository. The output of PointFinder [14] provided PubMed Identifiers for papers that described known point mutations that confer AMR. The drug class for AMR determinants were determined by using the associated paper and cross-referencing the AMR determinant name in the Comprehensive Antibiotic Resistance Database (CARD) v. 3.3.0 [15].

Supplementary Table 2. List of antimicrobial resistance genes and associated drug classifications.

**ARIBA AMR code**	**Gene ID**	**NCBI accession**	**Function**
aac_3__IId.1_EU022314_2	aac_3__IId	EU022314	Aminoglycoside
aac_3__IV.1_DQ241380_2	aac_3__IV	DQ241380	Aminoglycoside
aac_3__VIa.2_NC_009838_2	aac_3__VIa	NC_009838	Aminoglycoside
aadA1.2_FJ591054_2	aadA1	FJ591054	Aminoglycoside
aadA1.3_JQ414041_2	aadA1	JQ414041	Aminoglycoside
aadA1.4_JQ480156_2	aadA1	JQ480156	Aminoglycoside
aadA1.5_JX185132_2	aadA1	JX185132	Aminoglycoside
aadA17.1_FJ460181_2	aadA17	FJ460181	Aminoglycoside
aadA2.2_JQ364967_2	aadA2	JQ364967	Aminoglycoside
aadA22.1_AM261837_2	aadA22	AM261837	Aminoglycoside
aadA24.1_DQ677333_2	aadA24	DQ677333	Aminoglycoside
aadA2b.1_D43625_2	aadA2b	D43625	Aminoglycoside
aadA5.1_AF137361_2	aadA5	AF137361	Aminoglycoside
aadA8b.2_AM040708_2	aadA8b	AM040708	Aminoglycoside

aph_3____Ib.5_AF321551_2	aph_3____Ib	AF321551	Aminoglycoside
aph_3___Ia.1_V00359_2	aph_3___Ia	V00359	Aminoglycoside
aph_3___Ia.9_EU722351_2	aph_3___Ia	EU722351	Aminoglycoside
aph_4__Ia.1_V01499_2	aph_4__Ia	V01499	Aminoglycoside
aph_6__Id.1_M28829_2	aph_6__Id	M28829	Aminoglycoside
aph_6__Id.4_CP000971_2	aph_6__Id	CP000971	Aminoglycoside
ARR_2.1_HQ141279_2	ARR_2	HQ141279	Rifamycin
blaCARB_2.1_M69058_2	blaCARB_2	M69058	Beta-lactam
blaCMY_2.1_X91840_2	blaCMY_2	X91840	Beta-lactam
blaCTX_M_27.1_AY156923_2	blaCTX_M_27	AY156923	Beta-lactam
blaCTX_M_55.1_DQ810789_2	blaCTX_M_55	DQ810789	Beta-lactam
blaLAP_2.1_EU159120_2	blaLAP_2	EU159120	Beta-lactam
blaOXA_1.1_HQ170510_2	blaOXA_1	HQ170510	Beta-lactam
blaOXA_10.1_J03427_2	blaOXA_10	J03427	Beta-lactam
blaTEM_1A.1_HM749966_2	blaTEM_1A	HM749966	Beta-lactam
blaTEM_1B.1_AY458016_2	blaTEM_1B	AY458016	Beta-lactam
blaTEM_1C.1_FJ560503_2	blaTEM_1C	FJ560503	Beta-lactam
blaTEM_1D.1_AF188200_2	blaTEM_1D	AF188200	Beta-lactam
blaTEM_220.1_KM998962_2	blaTEM_220	KM998962	Beta-lactam
blaTEM_30.1_AJ437107_2	blaTEM_30	AJ437107	Beta-lactam
blaTEM_40.1_FR717535_2	blaTEM_40	FR717535	Beta-lactam
blaTEM_99.1_AF397066_2	blaTEM_99	AF397066	Beta-lactam
catA1.1_V00622_2	catA1	V00622	Chloramphenicol
catA2.1_X53796_2	catA2	X53796	Chloramphenicol
catB3.2_U13880_2	catB3	U13880	Chloramphenicol
cmlA1.1_M64556_2	cmlA1	M64556	Chloramphenicol
dfrA1.8_X00926_2	dfrA1	X00926	Trimethoprim
dfrA1.9_AJ238350_2	dfrA1	AJ238350	Trimethoprim

dfrA12.8_AM040708_2	dfrA12	AM040708	Trimethoprim
dfrA14.1_KF921535_2	dfrA14	KF921535	Trimethoprim
dfrA14.4_AF393510_2	dfrA14	AF393510	Trimethoprim
dfrA15.2_AF221900_2	dfrA15	AF221900	Trimethoprim
dfrA16.2_AF174129_2	dfrA16	AF174129	Trimethoprim
dfrA17.1_FJ460238_2	dfrA17	FJ460238	Trimethoprim
dfrA36.1_CP038791_2	dfrA36	CP038791	Trimethoprim
dfrA5.1_X12868_2	dfrA5	X12868	Trimethoprim
dfrA7.1_AB161450_2	dfrA7	AB161450	Trimethoprim
dfrA8.1_U10186_2	dfrA8	U10186	Trimethoprim
dfrB1.1_U36276_2	dfrB1	U36276	Trimethoprim
dfrB4.1_AJ429132_2	dfrB4	AJ429132	Trimethoprim
erm_42_.1_FR734406_2	erm_42	FR734406	Multidrug
floR.2_AF118107_2	floR	AF118107	Chloramphenicol
fosA7.1_LAPJ01000014_2	fosA7	LAPJ01000014	Fosfomycin
lnu_F_.1_EU118119_2	lnu_F	EU118119	Lincosamide
lnu_G_.1_KX470419_2	lnu_G	KX470419	Lincosamide
mcr_1.26.1_NG_068217_2	mcr_1	NG_068217	Colistin
mef_B_.1_FJ196385_2	mef_B	FJ196385	Macrolide
mph_A_.1_D16251_2	mph_A	D16251	Macrolide
mph_B_.1_D85892_2	mph_B	D85892	Macrolide
qnrB19.1_EU432277_2	qnrB19	EU432277	Quinolone
qnrB7.1_EU043311_2	qnrB7	EU043311	Quinolone
qnrS1.1_AB187515_2	qnrS1	AB187515	Quinolone
qnrS13.1_LUYD01000008_2	qnrS13	LUYD01000008	Quinolone
qnrS4.1_FJ418153_2	qnrS4	FJ418153	Quinolone
sul1.2_U12338_2	sul1	U12338	Sulfonamide
sul1.39_AY522923_2	sul1	AY522923	Sulfonamide

sul1.9_AY963803_2	sul1	AY963803	Sulfonamide
sul2.2_AY034138_2	sul2	AY034138	Sulfonamide
sul2.3_HQ840942_2	sul2	HQ840942	Sulfonamide
sul2.6_FN995456_2	sul2	FN995456	Sulfonamide
sul3.2_AJ459418_2	sul3	AJ459418	Sulfonamide
tet_A_.4_AJ517790_2	tet_A	AJ517790	Tetracycline
tet_A_.6_AF534183_2	tet_A	AF534183	Tetracycline
tet_B_.1_AP000342_2	tet_B	AP000342	Tetracycline
tet_B_.2_AF326777_2	tet_B	AF326777	Tetracycline
tet_C_.3_AF055345_2	tet_C	AF055345	Tetracycline
tet_M_.5_U58985_2	tet_M	U58985	Tetracycline
tet_M_.8_X04388_2	tet_M	X04388	Tetracycline

Supplementary Table 3. List of point mutations conferring AMR and associated drug classifications.

**Point mutation**	**Amino acid change**	**Resistance**	**PMID number**	**Function**
23S r.754G>A	G -> A	Erythromycin, Telithromycin	10027979	Macrolide
gyrA p.S83L	TCG -> TTG	Nalidixic acid, Ciprofloxacin	8891148	Quinolone
parC p.S57T	AGC -> ACC	Nalidixic acid, Ciprofloxacin	14510643	Quinolone
gyrA p.D87N	GAC -> AAC	Nalidixic acid, Ciprofloxacin	12654733	Quinolone
parC p.S80I	AGC -> ATC	Nalidixic acid, Ciprofloxacin	8851598	Quinolone
parE p.I355T	ATC -> ACC	Nalidixic acid, Ciprofloxacin	28598203	Quinolone

parE p.S458T	TCG -> ACG	Nalidixic acid, Ciprofloxacin	14506034	Quinolone
parC p.E84K	GAA -> AAA	Nalidixic acid, Ciprofloxacin	8524852	Quinolone
23S r.2032T>C	T -> C	Linezolid	10986233	Oxazolidinone
23S r.2058T>G	T -> G	Erythromycin, Azithromycin, Telithromycin	15616307	Macrolide

Supplementary Table 4. List of the unknown sequence type (ST) IDs and their corresponding combinations of the Achtman seven gene allele scheme.

adk	*fumC*	*gyrB*	*icd*	*mdh*	*purA*	*recA*	**Novel_ST**
adk(6)	fumC(11)	gyrB(4)	icd(~876)	mdh(7)	purA(8)	recA(6)	Novel 1
adk(83)	fumC(186)	gyrB(136)	icd(12)	mdh(1)	purA(2)	recA(2)	Novel 2
adk(~6)	fumC(6)	gyrB(5)	icd(10)	mdh(9)	purA(8)	recA(6)	Novel 3
adk(6)	fumC(19)	gyrB(3)	icd(18)	mdh(9)	purA(13,13)	recA(156)	Novel 4
adk(~20)	fumC(45)	gyrB(41)	icd(43)	mdh(5)	purA(32)	recA(2)	Novel 5
adk(6)	fumC(4)	gyrB(33)	icd(132)	mdh(20)	purA(~186)	recA(7)	Novel 6
adk(6)	fumC(23)	gyrB(608)	icd(16)	mdh(27)	purA(35)	recA(6)	Novel 7
adk(1065)	fumC(11)	gyrB(4)	icd(8)	mdh(8)	purA(8)	recA(2)	Novel 8
adk(6)	fumC(4)	gyrB(5)	icd(18)	mdh(~11)	purA(8)	recA(14)	Novel 9
adk(10)	fumC(11)	gyrB(4)	icd(8)	mdh(8)	purA(382)	recA(369)	Novel 10
adk(1)	fumC(251)	gyrB(207)	icd(25)	mdh(180)	purA(5)	recA(~19)	Novel 11
adk(1063?)	fumC(4)	gyrB(3)	icd(16)	mdh(11)	purA(8)	recA(6)	Novel 12
adk(6)	fumC(65)	gyrB(32)	icd(~26)	mdh(11)	purA(8)	recA(2)	Novel 13
adk(6)	fumC(65)	gyrB(33)	icd(16)	mdh(7)	purA(8)	recA(6)	Novel 14
adk(6)	fumC(4)	gyrB(4)	icd(16)	mdh(24)	purA(~8)	recA(14)	Novel 15
adk(6)	fumC(~11)	gyrB(4)	icd(8)	mdh(8)	purA(8)	recA(2)	Novel 16
adk(10)	fumC(11)	gyrB(4)	icd(8)	mdh(616)	purA(8)	recA(2)	Novel 17
adk(6)	fumC(~93)	gyrB(26)	icd(82)	mdh(1)	purA(2)	recA(2)	Novel 18
adk(6)	fumC(19)	gyrB(22)	icd(26)	mdh(11)	purA(8)	recA(2)	Novel 19
adk(6)	fumC(95)	gyrB(15)	icd(18)	mdh(9)	purA(8)	recA(6)	Novel 20
adk(224)	fumC(4)	gyrB(54)	icd(247)	mdh(61)	purA(1)	recA(7)	Novel 21
adk(876?)	fumC(45)	gyrB(41)	icd(43)	mdh(5)	purA(32)	recA(2)	Novel 22
adk(10)	fumC(~11)	gyrB(57)	icd(8)	mdh(7)	purA(18)	recA(6)	Novel 23
adk(8)	fumC(107)	gyrB(4)	icd(8)	mdh(8)	purA(8)	recA(2)	Novel 24
adk(6)	fumC(~29)	gyrB(32)	icd(16)	mdh(9)	purA(8)	recA(2)	Novel 25
adk(6)	fumC(6)	gyrB(5)	icd(85)	mdh(9)	purA(8)	recA(7)	Novel 26
adk(6)	fumC(6)	gyrB(~33)	icd(1)	mdh(24)	purA(7)	recA(7)	Novel 27

adk(~6)	fumC(4)	gyrB(1)	icd(95)	mdh(69)	purA(8)	recA(20)	Novel 28
adk(10)	fumC(~594)	gyrB(5)	icd(8)	mdh(7)	purA(8)	recA(6)	Novel 29
adk(6)	fumC(6)	gyrB(5)	icd(10)	mdh(20)	purA(~23)	recA(6)	Novel 30
adk(6)	fumC(6)	gyrB(33)	icd(1084)	mdh(24)	purA(8)	recA(7)	Novel 31
adk(6)	fumC(~19)	gyrB(3)	icd(18)	mdh(9)	purA(13)	recA(156)	Novel 32
adk(10)	fumC(~7)	gyrB(4)	icd(8)	mdh(12)	purA(8)	recA(2)	Novel 33
adk(87)	fumC(~958)	gyrB(~186)	icd(~1123)	mdh(~42)	purA(162)	recA(~440)	Novel 34
adk(10)	fumC(7?)	gyrB(4)	icd(8)	mdh(12)	purA(8)	recA(2)	Novel 35
adk(20)	fumC(~1466)	gyrB(41)	icd(43)	mdh(~5)	purA(548)	recA(46)	Novel 36
adk(10)	fumC(11)	gyrB(4)	icd(8)	mdh(8)	purA(219)	recA(2)	Novel 37
adk(35)	fumC(50)	gyrB(157)	icd(49)	mdh(37)	purA(41)	recA(191)	Novel 38
adk(9)	fumC(~6)	gyrB(33)	icd(131)	mdh(24)	purA(8)	recA(7)	Novel 39
adk(111)	fumC(11)	gyrB(57)	icd(8)	mdh(~7)	purA(18)	recA(6)	Novel 40
adk(6)	fumC(19)	gyrB(15)	icd(56)	mdh(9)	purA(2)	recA(2)	Novel 41
adk(1077?)	fumC(4)	gyrB(33)	icd(16)	mdh(11)	purA(8)	recA(6)	Novel 42
adk(10)	fumC(4)	gyrB(4)	icd(1)	mdh(12)	purA(400)	recA(2)	Novel 43
adk(82)	fumC(~1220)	gyrB(607)	icd(79)	mdh(~541)	purA(~425)	recA(~674)	Novel 44
adk(10)	fumC(11)	gyrB(4)	icd(8)	mdh(~8)	purA(8)	recA(2)	Novel 45
adk(64)	fumC(7)	gyrB(1)	icd(1)	mdh(8)	purA(~8)	recA(6)	Novel 46
adk(6)	fumC(29)	gyrB(14)	icd(18)	mdh(9)	purA(26)	recA(7)	Novel 47
adk(9)	fumC(6)	gyrB(15)	icd(131)	mdh(~24)	purA(7)	recA(7)	Novel 48
adk(~31)	fumC(~443)	gyrB(54)	icd(10)	mdh(1)	purA(12)	recA(257)	Novel 49
adk(6)	fumC(11)	gyrB(4)	icd(10)	mdh(7)	purA(8)	recA(~7)	Novel 50
adk(10)	fumC(11)	gyrB(57)	icd(~8)	mdh(7)	purA(18)	recA(6)	Novel 51
adk(8)	fumC(11)	gyrB(4)	icd(8)	mdh(41)	purA(~8)	recA(2)	Novel 52
adk(154)	fumC(36)	gyrB(207)	icd(~63)	mdh(180)	purA(5)	recA(4)	Novel 53
adk(1059?)	fumC(11)	gyrB(57)	icd(140)	mdh(7)	purA(175)	recA(6)	Novel 54

Supplementary Table 5. Presence of most prevalent sequence types (STs) in this dataset across all genomes and within all samples.

Most prevalent STs	Presence across all genomes	Presence within all samples
ST10	6.47%, n = 69 genomes	11.2%, n = 45 samples
ST117	3.66%, n = 39 genomes	7.73%, n = 31 samples
ST101	3.28%, n = 35 genomes	5.99%, n = 24 samples
ST155	2.44%, n = 26 genomes	4.49%, n = 18 samples
ST216	2.16%, n = 23 genomes	3.24%, n = 13 samples
ST5474	2.16%, n = 23 genomes	2.49%, n = 10 samples

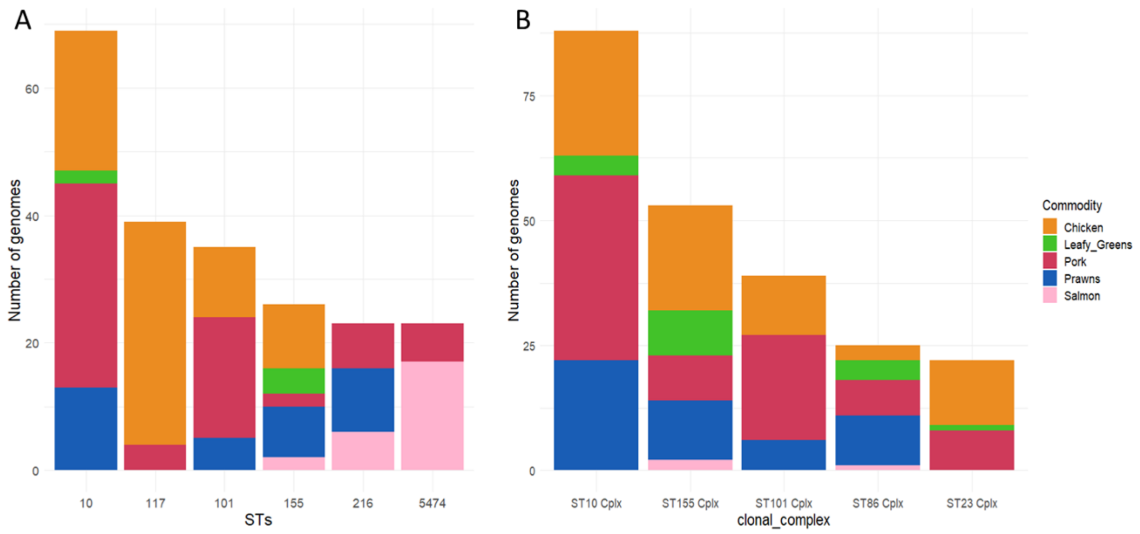

Supplementary Figure 1. (A) The number of *E. coli* genomes found for the most prevalent STs and (B) for the most prevalent clonal complexes in this dataset, categorised by food commodity.

Supplementary Table 6. List of *E. coli* STs specific to a food commodity; novel STs are defined in Supplementary Table 3.

Commodity	STs specific to the food commodity	Number of samples with a unique ST detected

Chicken	1 Novel, 10 Novel, 1056, 106, 1140, 115, 1158, 1163, 1170, 1172, 1276, 1286, 1304, 135, 140, 1483, 1485, 1551, 1594, 1611, 1640, 1737, 174, 1775, 2 Novel, 2001, 2040, 2171, 2280, 2473, 2520, 2599, 2722, 2772, 2792, 3 Novel, 3006, 3090, 3107, 3234, 350, 352, 354, 3549, 359, 373, 38, 4 Novel, 40, 442, 457, 4674, 4993, 5 Novel, 5203, 533, 5375, 539, 5796, 6 Novel, 6286, 6635, 665, 6664, 7 Novel, 7013, 744, 752, 8 Novel, 8611, 8874, 9 Novel, 95	103
Pork	1020, 1125, 1204, 1244, 1248, 13, 1308, 1431, 164, 165, 1718, 1972, 201, 2035, 21 Novel, 2178, 22 Novel, 23 Novel, 25 Novel, 26 Novel, 2628, 27 Novel, 28 Novel, 29 Novel, 30 Novel, 31 Novel, 32 Novel, 33 Novel, 34 Novel, 345, 35 Novel, 361, 410, 4247, 446, 453, 4580, 4704, 472, 4994, 5082, 5409, 542, 567, 6150, 625, 635, 6745, 6778, 710, 75, 7940, 898, 90, 971	67
Leafy Greens	10176, 11 Novel, 1167, 12 Novel, 13 Novel, 130, 14 Novel, 15 Novel, 16 Novel, 1629, 17 Novel, 1723, 1730, 18 Novel, 1858, 19 Novel, 20 Novel, 2005, 219, 2522, 2602, 28, 3167, 34, 3568, 3576, 4118, 4197, 452, 5156, 5328, 5478, 56, 5614, 5765, 6188, 642, 6422, 6603, 906, 937	43
Salmon	1115, 3604, 480, 536, 54 Novel, 6163, 720, 8763	13
Prawns	1, 10512, 1196, 1246, 181, 1823, 197, 2160, 2169, 226, 2521, 2825, 2954, 3232, 3489, 357, 3580, 36 Novel, 37 Novel, 38 Novel, 3863, 39 Novel, 40 Novel, 41 Novel, 42 Novel, 4221, 43 Novel, 4377, 44 Novel, 45 Novel, 46 Novel, 4682, 4684, 47 Novel, 48 Novel, 49 Novel, 50 Novel, 51 Novel, 52 Novel, 5295, 53 Novel, 5359, 5523, 6027, 6241, 683, 8262, 8576	52

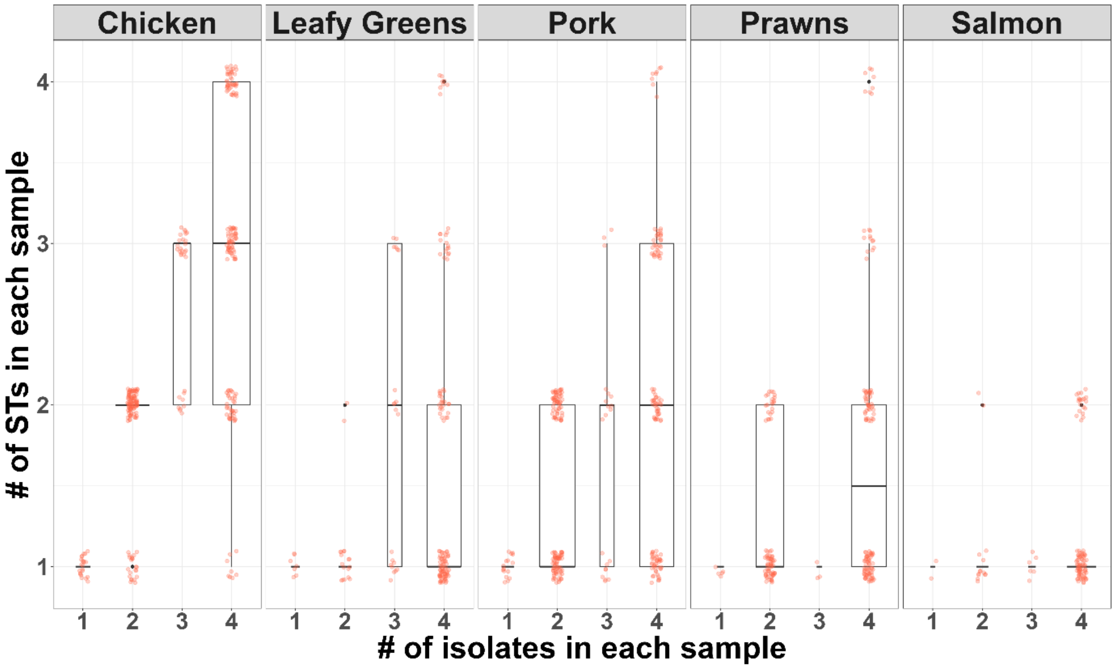

<u>Supplementary Figure 2.</u> Boxplots of the 401 retail chicken, pork, prawns, salmon, and leafy greens samples. The Y axis shows the number of sequence types (STs) when up to four *E. coli* isolates in each sample were sequenced. Each red dot represents a sample. The box shows the interquartile range of the number of STs in each sample. The whiskers are calculated as 1.5 times the interquartile range and the black dots outside the boxplot whiskers are outlier samples.

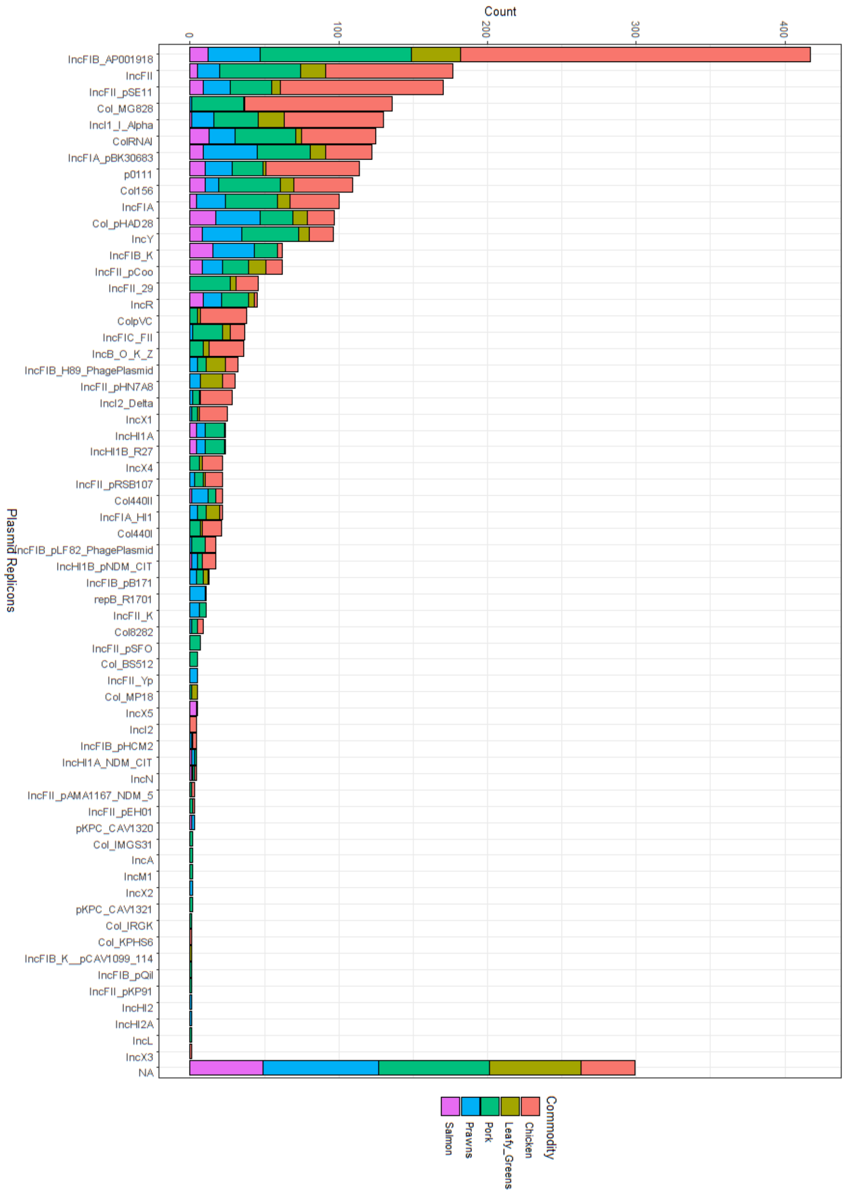

<u>Supplementary Figure 3.</u> Distribution of plasmid replicons identified within the 1,067 *E. coli* across the food commodities within the 401 food samples.

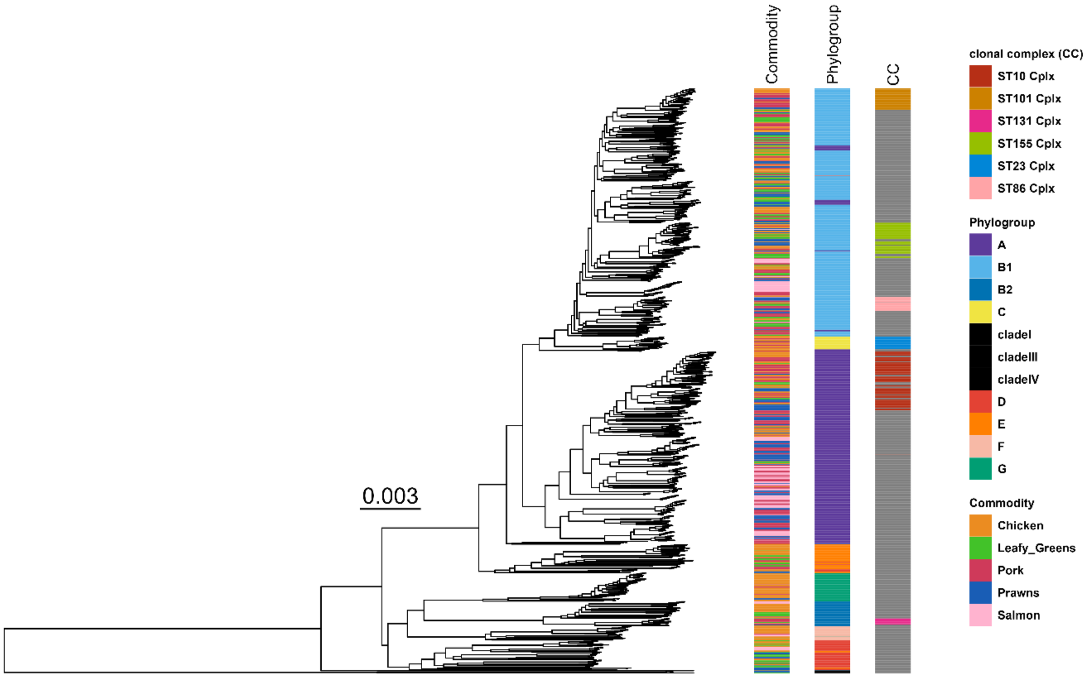

Supplementary Figure 4. Phylonium midpoint rooted phylogenetic tree of 1,067 *E. coli* genomes from five retail food commodities with a tree scale estimating 0.003 nucleotide substitutions per site. Commodity (left) and phylogroup (middle) and clonal complex (right) are mapped onto the tree.

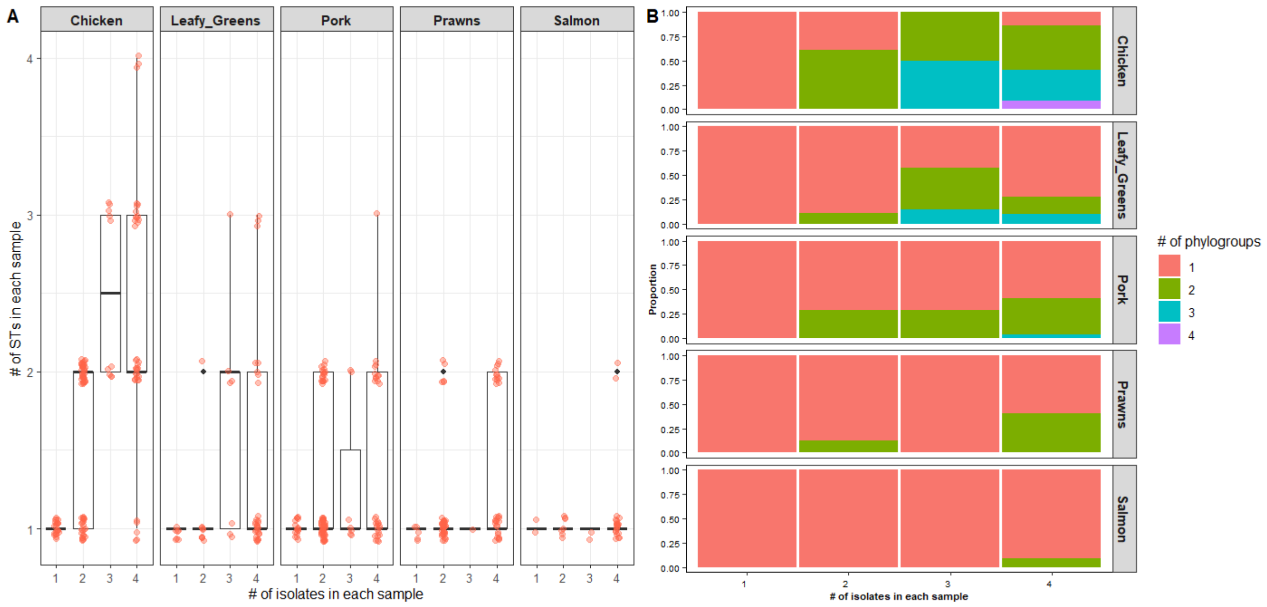

Supplementary Figure 5. (A) Boxplots of the 1,067 *E. coli*, showing the number of phylogroups recovered in all 401 food samples when up to four *E. coli* isolates per sample were selected, separated by food commodity and (B) Stacked bar plots of the 1,067 *E. coli,* showing the proportion of phylogroups recovered when up to four *E. coli* isolates per sample were selected.

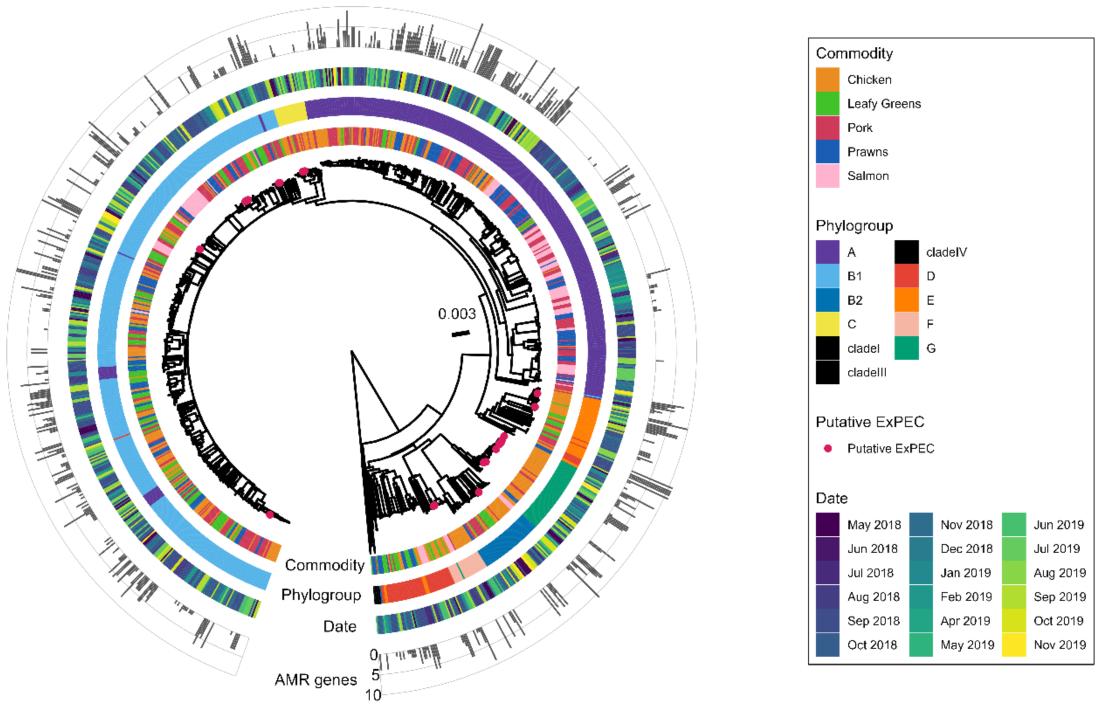

Supplementary Figure 6. Phylonium midpoint rooted phylogenetic tree of the collection of 1,067 *E. coli* genomes isolated from retail chicken, pork, prawns, salmon, and leafy greens with a tree scale estimating 0.003 nucleotide substitutions per site. Tree tips are labelled red if they are putative extra-intestinal pathogenic *E. coli* (ExPEC). The commodity (first inner ring), phylogroup (second inner ring), date collected (third inner ring), and the total number of AMR determinants are mapped onto the tree (outer ring).

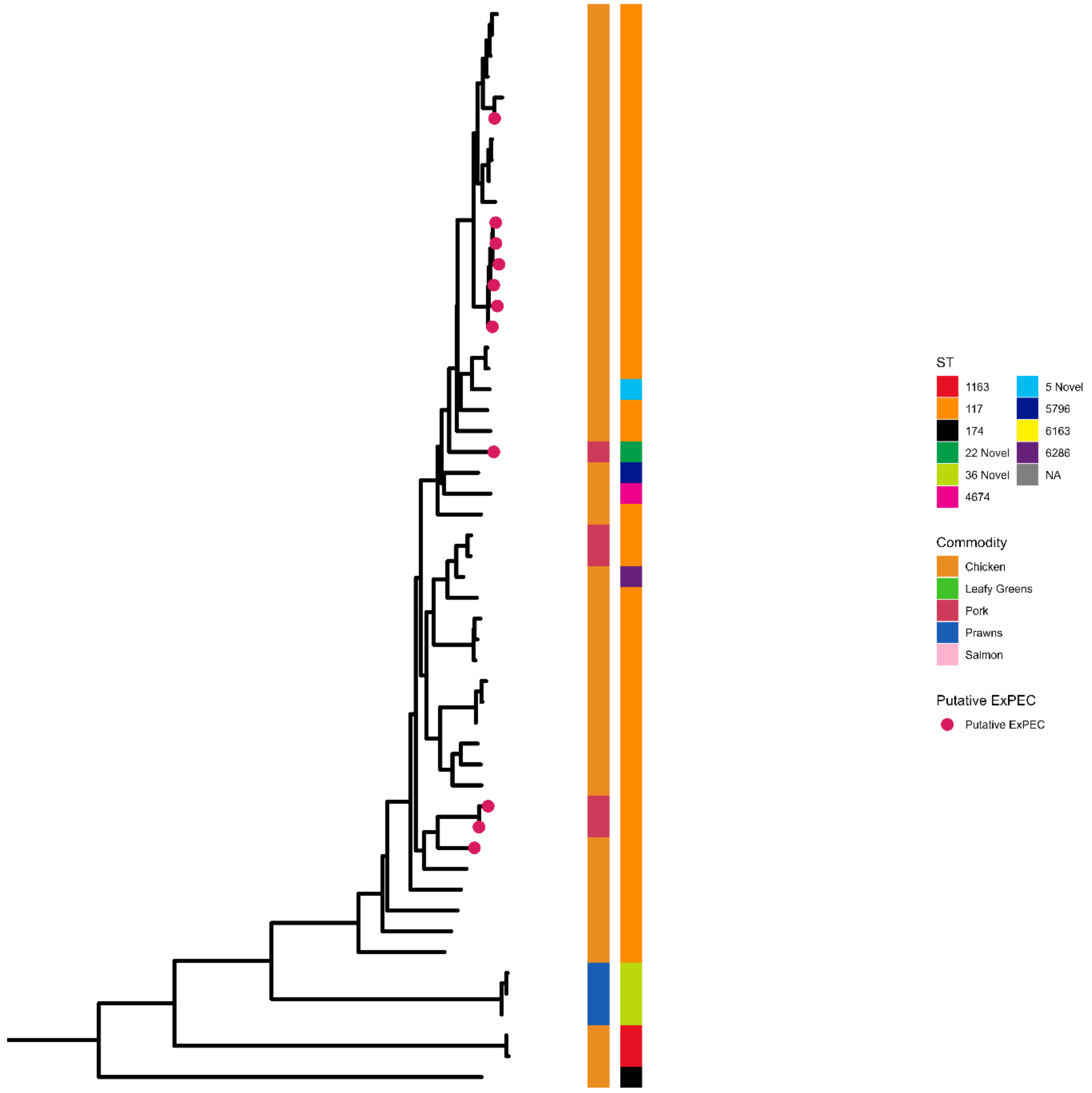

<u>Supplementary Figure 7.</u> A clade of the Phylonium midpoint rooted phylogenetic tree of the collection of 1,067 *E. coli* genomes isolated from retail chicken, pork, prawns, salmon, and leafy greens with a tree scale estimating 0.003 nucleotide substitutions per site, highlighting phylogroup G. Tree tips are labelled red if they are putative extra-intestinal pathogenic *E. coli* (ExPEC). The commodity (first ring) and sequence type (second ring) are labelled.

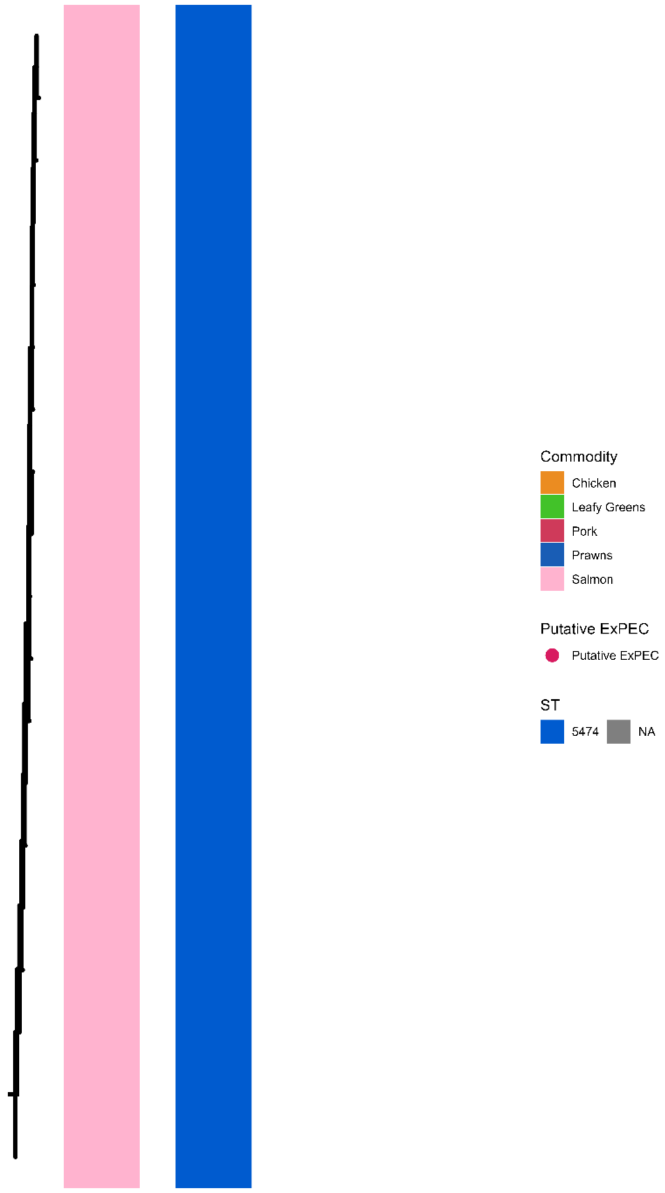

Supplementary Figure 8. A clade of the Phylonium midpoint rooted phylogenetic tree of the collection of 1,067 *E. coli* genomes isolated from retail chicken, pork, prawns, salmon, and leafy greens with a tree scale estimating 0.003 nucleotide substitutions per site, highlighting a subset of phylogroup B1 with a cluster of *E. coli* genomes isolated from salmon. Tree tips are labelled red if they are putative extra-intestinal pathogenic *E. coli* (ExPEC). The commodity (first ring) and phylogroup (second ring) are labelled.

Supplementary Table 7. Number of *E. coli* genomes within phylogroups within the 1,067 food-derived genomes

Phylogroups (n genomes)	Number of genomes
A	376
B1	432

B2	46
C	23
D	57
E	50
F	25
G	53
Cryptic clade I	1
Cryptic clade III	1
Cryptic clade IV	3

References

1.Li H, Durbin R. Fast and accurate short read alignment with Burrows-Wheeler transform. *Bioinformatics* 2009;25(14):1754-1760.

2.Li H, Handsaker B, Wysoker A, Fennell T, Ruan J, Homer N, Marth G, Abecasis G, Durbin R, Genome Project Data P. The Sequence Alignment/Map format and SAMtools. *Bioinformatics* 2009;25(16):2078-2079.

3.Bloomfield SJ, Zomer AL, O'Grady J, Kay GL, Wain J, Janecko N, Palau R, Mather AE. Determination and quantification of microbial communities and antimicrobial resistance on food through host DNA-depleted metagenomics. *Food Microbiology* 2023;110:104162.

4.Simonsen M, Mailund T, Pedersen CNS. Rapid Neighbour-Joining. *Algorithms in Bioinformatics* 2008;5251:113-122.

5.R Core Team. 2022. R: A language and environment for statistical computing. R. https://www.R-project.org/ [accessed 15/04/2025].

6.RStudio Team. 2020. RStudio: Integrated Development for R. http://www.rstudio.com/ [accessed 15/04/2025].

7.Yu G. Using ggtree to Visualize Data on Tree-Like Structures. *Current Protocols in Bioinformatics* 2020;69(1):e96.

8.Paradis E, Schliep K. ape 5.0: an environment for modern phylogenetics and evolutionary analyses in R. *Bioinformatics* 2019;35(3):526-528.

9.Revell LJ. phytools: an R package for phylogenetic comparative biology (and other things). *Methods in Ecology and Evolution* 2012;3(2):217-223.

10.Campitelli E. 2022. ggnewscale: Multiple Fill and Colour Scales in 'ggplot2'. https://doi.org/10.5281/zenodo.2543762 [accessed 15/04/2025].

11.Wickham H. 2016. ggplot2: Elegant Graphics for Data Analysis. https://ggplot2.tidyverse.org [accessed 11/08/2025].

12.Hunt M, Mather AE, Sanchez-Buso L, Page AJ, Parkhill J, Keane JA, Harris SR. ARIBA: rapid antimicrobial resistance genotyping directly from sequencing reads. *Microbial Genomics* 2017;3(10).

13.Bortolaia V, Kaas RS, Ruppe E, Roberts MC, Schwarz S, Cattoir V, Philippon A, Allesoe RL, Rebelo AR, Florensa AF, Fagelhauer L, Chakraborty T, Neumann B, Werner G, Bender JK, Stingl K, Nguyen M, Coppens J, Xavier BB, Malhotra-Kumar S, Westh H, Pinholt M, Anjum MF, Duggett NA, Kempf I, Nykasenoja S, Olkkola S, Wieczorek K, Amaro A, Clemente L, Mossong J, Losch S, Ragimbeau C, Lund O, Aarestrup FM. ResFinder 4.0 for predictions of phenotypes from genotypes. *Journal of Antimicrobial Chemotherapy* 2020;75(12):3491-3500.

14.Zankari E, Allesøe R, Joensen KG, Cavaco LM, Lund O, Aarestrup FM. PointFinder: a novel web tool for WGS-based detection of antimicrobial resistance associated with chromosomal point mutations in bacterial pathogens. *Journal of Antimicrobial Chemotherapy* 2017;72(10):2764-2768.

15.McArthur AG, Waglechner N, Nizam F, Yan A, Azad MA, Baylay AJ, Bhullar K, Canova MJ, De Pascale G, Ejim L, Kalan L, King AM, Koteva K, Morar M, Mulvey MR, O'Brien JS, Pawlowski AC, Piddock LJ, Spanogiannopoulos P, Sutherland AD, Tang I, Taylor PL, Thaker M, Wang W, Yan M, Yu T, Wright GD. The comprehensive antibiotic resistance database. *Antimicrobial Agents and Chemotherapy* 2013;57(7):3348-3357.
